# Comparison of allele frequencies of *Plasmodium falciparum* merozoite antigens in malaria infections sampled in different years in a Kenyan population

**DOI:** 10.1186/s12936-016-1304-8

**Published:** 2016-05-06

**Authors:** Lynette Isabella Ochola-Oyier, John Okombo, Njoroge Wagatua, Jacob Ochieng, Kevin K. Tetteh, Greg Fegan, Philip Bejon, Kevin Marsh

**Affiliations:** KEMRI-Wellcome Trust Collaborative Programme, P.O. Box 230, Kilifi, 80108 Kenya; Department of Immunology and Infection, Faculty of Infectious and Tropical Diseases, London School of Hygiene & Tropical Medicine, London, UK

**Keywords:** Rh5, Merozoite, *Plasmodium*, Malaria

## Abstract

**Background:**

*Plasmodium falciparum* merozoite antigens elicit antibody responses in malaria-endemic populations, some of which are clinically protective, which is one of the reasons why merozoite antigens are the focus of malaria vaccine development efforts. Polymorphisms in several merozoite antigen-encoding genes are thought to arise as a result of selection by the human immune system.

**Methods:**

The allele frequency distribution of 15 merozoite antigens over a two-year period, 2007 and 2008, was examined in parasites obtained from children with uncomplicated malaria. In the same population, allele frequency changes pre- and post-anti-malarial treatment were also examined. Any gene which showed a significant shift in allele frequencies was also assessed longitudinally in asymptomatic and complicated malaria infections.

**Results:**

Fluctuating allele frequencies were identified in codons 147 and 148 of reticulocyte-binding homologue (Rh) 5, with a shift from HD to YH haplotypes over the two-year period in uncomplicated malaria infections. However, in both the asymptomatic and complicated malaria infections YH was the dominant and stable haplotype over the two-year and ten-year periods, respectively. A logistic regression analysis of all three malaria infection populations between 2007 and 2009 revealed, that the chance of being infected with the HD haplotype decreased with time from 2007 to 2009 and increased in the uncomplicated and asymptomatic infections.

**Conclusion:**

Rh5 codons 147 and 148 showed heterogeneity at both an individual and population level and may be under some degree of immune selection.

**Electronic supplementary material:**

The online version of this article (doi:10.1186/s12936-016-1304-8) contains supplementary material, which is available to authorized users.

## Background

Genes involved in immune interactions tend to contain adaptive variation that is maintained by balancing selection [1, 2]. In *Plasmodium falciparum* balancing selection is evidenced by the presence of multiple polymorphisms in several merozoite antigens, such as merozoite surface protein-1 (MSP-1) [3], apical membrane antigen-1 (AMA-1) [4], erythrocyte binding antigen-175 (EBA-175) [5], MSP3 [6], MSP Duffy Binding Ligand-1 (DBL-1) [7], MSPDBL2 [8], reticulocyte binding homologues Rh2a and Rh2b [9, 10].

Since merozoite proteins are targets of host immunity [10–13] polymorphisms are thought to be maintained by selection from host immune responses. There is evidence to suggest polymorphisms could result in primarily allele-specific immune responses [6, 14, 15]. Therefore, an individual may resist infection to one parasite allele for which they have pre-existing immunity but not to a heterologous allele that they have not yet encountered. Initially over time, rare alleles attracting less ‘immune attention’ would be expected to progressively proliferate while dominant alleles are cleared by the immunity they induce [16]. Thus, the polymorphisms are potentially maintained by a mechanism of frequency-dependent selection. Allele-specific immunity may limit the efficacy of vaccines developed against the merozoite blood stage antigens [17–19].

Stable balancing selection predicts constant intermediate allele frequencies over long periods [20], which change more slowly over time than they would under neutral genetic drift (2). This has been demonstrated in merozoite antigens in longitudinal studies, which primarily assessed repeat regions in MSP1 and MSP2 alleles [16, 21–24]. Similar observations were made with other merozoite antigens SURFIN4.1 [25], SURFIN4.2 [26] and GLURP [27]. In contrast, under fluctuating selection due to spatial and temporal variation, allele frequencies dynamically change over time becoming common when advantageous and rare when disadvantageous [28, 29], and allele frequencies should change more quickly over time than they would under neutral genetic drift [2]. There are a few cases where some merozoite antigen polymorphisms fluctuate in frequency over time [16, 21, 30, 31].

The basis of the frequency-dependent selection operating on merozoite antigens is not entirely understood. It is likely to reflect both the function of merozoite antigens as invasion receptors (erythrocyte-merozoite interactions) and the impact of the immune system on the merozoite. Understanding the distribution of merozoite antigen polymorphisms in natural parasite populations can inform the vaccine design process. The main aim of this study was to analyse the allele frequency distribution of 15 merozoite antigens in uncomplicated malaria infections from children under 5 years of age in an endemic area in Kilifi, Kenya over a two-year period, in order to identify single nucleotide polymorphisms (SNPs) under selection. Any significantly fluctuating SNPs were then investigated in asymptomatic and complicated malaria infections. Two SNPs (codons 147 and 148) in Rh5 were identified, which significantly fluctuated over the two-year period in uncomplicated malaria infections and remained stable in the asymptomatic and complicated malaria infections. Within the same population (the children under 5 years of age with uncomplicated malaria) the changes in allele frequencies pre- and post-anti-malarial treatment were also studied. Rh5 codons 147 and 148 were more likely to change during the course of an infection pre- and post-drug treatment. Additionally, the extent of genetic diversity was examined within and between the genes usling linkage disequilibrium (LD).

## Methods

### Longitudinal *Plasmodium falciparum* population samples

Merozoite antigenic diversity was examined by genotyping 15 genes (see Additional file 1) in 100 parasites obtained from children aged up to five years with uncomplicated malaria, between 2007 (n = 62) and 2008 (n = 38), recruited into a randomized, controlled trial of dihydroartemisinin-piperaquine versus artemether–lumefantrine in Pingilikani sub-location, Kilifi County (Trial registration: Controlled-Trials.com ISRCTN88705995) [32]. Any gene that showed a significant temporal change in allele frequency was also sequenced in three other parasite populations (to determine if the change was observed in other malaria infection populations): (1) asymptomatic malaria infections from an annual community survey of parasite-positive children aged up to 12 years; 266 cross-sectional venous bleed samples were obtained before the malaria season in 2007 (n = 108), 2008 (n = 104) and 2009 (n = 54) in Junju sub-location, Kilifi County, Kenya. Junju and Pingilikani are generally high to moderate transmission areas, although transmission is falling in Kilifi County as a whole [33]. Pingilikani and Junju neighbour each other and the dispensaries are at least 10 km apart; (2) 300 isolates taken from parasite-positive children aged up to 12 years admitted to the Kilifi District Hospital (KDH) between 2007 and 2009 (100 isolates each year). This was later extended to 100 samples each over a ten-year period from 2000 to 2010. These samples were defined as complicated malaria infections and were also representative of the parasite population in Kilifi County since KDH serves a population within an 891 km^2^ area of 15 administrative locations within the Kilifi Health and Demographic Surveillance System (KHDSS) [34]; and, (3) 11 genotypically distinct laboratory isolates obtained from geographically diverse *P. falciparum* sources (3D7, cloned from an airport malaria case in The Netherlands; RO33 and Palo Alto, from Africa; K1, Dd2, FCC2 and D10, V1/S from Southeast Asia; Wellcome, nominally from Africa but suspected to have been previously cross-contaminated by parasites of unknown source during culture; HB3 from Honduras and IT from Brazil). Parasite DNA was extracted from packed frozen erythrocytes using the QIAamp DNA Blood Mini Kit (QIAGEN, UK).

### Pre- and post-drug treatment *Plasmodium falciparum* samples

The uncomplicated malaria samples were obtained from the randomized, controlled drug trial described above. The pre-treatment sample (day 0) was obtained on recruitment of a child into the study. The children were followed up weekly until day 63 and finally on day 84, resulting in a post-treatment sample (range 19–84 days, mean number of days = 50) if a child was parasite slide-positive during a follow-up visit. The samples were previously genotyped using MSP1, MSP2 and glutamate-rich protein (GLURP) [32] and a large majority of the post-treatment samples were re-infections (about 64 %) and 8 % were recrudescent infections the remaining were indeterminate. Parasite genomic DNA was extracted from dried filter-paper blood spots using the boiling method described elsewhere [35]. Briefly, this involved snipping a small piece of the dried filter paper, fixing it in methanol and boiling at 100 °C in TB Elution buffer to extract the DNA. All the above studies were reviewed and approved by the Scientific Steering Committee and the Ethics Committee of the Kenya Medical Research Institute. Consent for study participation was obtained from the parents or legal guardian of the study participants.

### PCR and sequencing of polymorphic *Plasmodium falciparum* genes

The 15 merozoite antigens were amplified using High Fidelity Taq polymerase (Roche) (details of regions amplified and primers are shown in Additional file 1). PCR-generated products were visualized on 1 % agarose gels, purified using ethanol precipitation and directly sequenced using the PCR primers, additional sequencing primers, BIG DYE terminator chemistry v3.1 (Applied Biosystems, UK) and an ABI 3130xl capillary sequencer (Applied Biosystems, UK). Nucleotide positions which displayed a peak within a peak in the electropherogram were defined as a mixed base and excluded from further analysis. Sequences were assembled, edited and aligned using SeqMan and MegAlign (DNASTAR, Madison, WI, USA). SNPs were identified and using their corresponding amino acids, haplotypes were defined.

### Statistical analysis of longitudinal *Plasmodium falciparum* polymorphic genes

To analyse SNP frequency dynamics in the 15 merozoite genes, comparisons were conducted within the uncomplicated malaria parasite population for SNPs that showed a significant temporal shift (between 2007 and 2008) using the Fisher’s exact test. SNPs with a >5 % minor allele frequency (MAF) were translated into their corresponding amino acids, in order to define haplotypes, which were also analysed. Any gene which maintained significance after a Bonferroni correction for multiple comparisons [36] was analysed further for temporal allele frequency changes in a primarily asymptomatic malaria parasite population between 2007 and 2009, and in a complicated malaria parasite population obtained from KDH between 2007 and 2009. A logistic regression analysis was performed on all the three parasite populations, controlling for year, age and population. Each gene was then examined for allele frequency changes over a ten-year period in the KDH sample set. Within the ten-year complicated malaria infection samples, the homogeneity of the infected population was determined and investigated if there were any linear trends of location, age and severe malaria syndrome, coma, deep breathing, and anaemia [37] using the ‘tabodds’ command in Stata. All analyses were conducted using Stata v11 (StataCorp, TX, USA) and R. Using the available latitude and longitude co-ordinates, the Rh5 haplotypes from the uncomplicated (Pingilikani), asymptomatic (Junju) and complicated (KDH) malaria parasite populations were plotted on to the KHDSS map [34]. The spatial scan statistic (SaTScan) software [38] was then employed as in Bejon et al. [39], to identify significant haplotype clusters, using a Bernoulli (case–control) model to test for significant clusters between the two dominant haplotypes and a multinomial model to test between all the haplotypes in each of the parasite populations between 2007 and 2009.

### Statistical analysis of pre- and post-drug treatment *Plasmodium falciparum* polymorphic genes

The overall population proportions of merozoite gene allelic pairs which were the same and different pre- and post-treatment from the same individual were determined. The parasite pairs were defined using haplotypes consisting of the amino acid codes for genes (*rh1, rh5, eba140, ebl*-*1, msp1*_*42*_) with a maximum of six SNPs and in genes (*rh2a, rh2b, rh4, eba175, eba181, ama1, msp3, msp6, mspdbl1, mspdbl2*), containing greater than six SNPs we assessed individual SNPs. The number of haplotype changes per parasite pair, pre- and post-treatment, were determined for each gene. A non-parametric test (McNemar’s test) for matched paired data was then done to analyse the proportion of heterologous compared to homologous haplotypes at each locus. Mixed infections were excluded from the analysis. To determine whether SNPs were in LD, all possible pair-wise comparisons between SNPs across all the genes were performed using a pair-wise correlation analysis with a Bonferroni correction for multiple comparisons [36].

## Results

### Merozoite gene haplotype frequencies in the uncomplicated malaria infections between 2007 and 2008

Most haplotypes were present as stable frequencies over the two-year period. There were two dominant haplotypes for EBL1-QFFFF.VN and QLFLSKS, EBA181-NKSFN and NQSFN and EBA140-VSTK and INTK; the 3D7 reference EBA140 haplotype, INKK, was not observed in this population (see Additional file 2). The 5′ end of the MSP1 42 kDa sequence contained a 3-bp deletion which has not been described before (SNP4414-6), resulting in three stable and high frequency haplotypes, FKFFDD (3D7 reference), FKFFDN, FFFDD. MSP3 K1 and MSP6 3D7 were the prevalent and stable haplotypes. The MSPDBL1 and MSPDBL2 haplotypes were defined as previously described [40]. Shifts in haplotype frequencies were observed in MSPDBL1 from AHQIARY to ALTAIKY, and for MSPDBL2, AHQAIRY was the dominant haplotype in both years. The variation in the Rh family was characterized by assessing previously defined polymorphic regions (see Additional file 1) [41]. The C-terminal end of Rh1 included two dipeptide (HNQN) repeats. The common dipeptide repeats represented the 3D7 reference sequence, 4HNQN, which shifted to 3HN2QN in 2008 and 4HN2QN remained stable over the two years. Rh2a and Rh4 both contained as the stable and widespread genotype the 3D7 reference haplotypes, KAKQQR and (IHTNENNINN)(EHTNENNINN)(EHTNEKNINN)(EHANEKNIYN)(EHTNENNINY), respectively. In Rh2b, two previously described deletions, a 192-bp deletion (codon 2717) [42] and a 585-bp deletion (codon 2954) [42, 43] were identified. The 156-bp insertion, Q (codon 2795) and a 585-bp deletion (156insQ585del) was dominant and stable, while 156del585del and 192ins585del were only present at high frequency in 2007 and 2008, respectively. The 3D7 reference genotype, 156ins585ins, was not observed in this population. A shift was also observed in Rh5 from N(DYKNVDYKNV)HDY to N(DYKNVDYKNV)YHY. The previously described EBA175 6-bp deletion [5] was identified and distinct fluctuations in the haplotypes from EEKKSISENKKI to EKEKPISENKKK between 2007 and 2008 were observed. AMA1 was defined using the amino acids in cluster 1 loop of domain I (codons 196, 197, 199, 200, 201, 204, 206, 207) [44], DQRHFDKY (16 %) and NGRDLNEY (14 %) were the two high frequency haplotypes. Sequences for all 15 genes were submitted to GenBank and are available under the accession codes EBL1:KU525776–KU525801, MSP6:KU525802–KU525809, KU525811, KU525813–KU525817, KU525819, KU525822–KU525844, Rh2b:KU525845–KU525879, Rh5:KU525880–KU525986, Rh2a:KU525988–KU526029, MSPDBL1:KU526030–KU526063, EBA140:KU526064–KU526129, Rh1:KU526130–KU526176, MSPDBL2:KU526177–KU526235, EBA175:KU526236–KU526265, EBA181:KU526266–KU526296, Rh4:KU526297–KU526361, MSP1:KU526362–KU526442, Rh5_10yr_samples: KU526443–KU526857 and KU526859–KU526963, AMA1:KU526964–KU527018, MSP3:KU527019–KU527058.

### Genetic diversity pre- and post-treatment

Apart from *ama1, msp1, eba175, mspdbl2, mspdbl1* and *rh2b* SNPs, which were 100 % different parasite pairs, though the latter two genes included an analysis of three pairs, in the rest of the genes the number of parasite pairs that were the same were <50 %. However, Rh4 (68 %) and Rh5 (70 %) (see Additional file 3) contained a larger number of parasite pairs that were the same. Only Rh5 showed a significant (McNemar’s χ^2^ = 4, p = 0.046) change in haplotype frequencies with an increased odds (OR 1.3, 95 % CI 1–1.7) of different haplotype parasite pairs compared to the same pairs (Table 1) pre- and post-treatment.

**Table 1:** McNemar’s χ^2^ test of haplotype proportions from pre- and post-treatment parasite pairs

	Pre- and post-treatment				
	Pre	Post		McNemar’s χ^2^ (p value)	OR (95 % CI)
AMA1		DQRHFDKY	Other		
	DQRHFDKY	0	0		
	Other	2	8	2 (0.16)	ND
EBA140		VSTK	Other		
	VSTK	5	2		
	Other	3	5	0.2 (0.65)	0.9 (0.5–1.6)
EBA181		Q	K		
	Q	5	4		
	K	2	2	0.67 (0.41)	1.3 (0.7–2.4)
EBA175		EKEKPISENKKK	Other		
	EKEKPISENKKK				
	Other	No data			
EBL-1		QFFFF.VN	Other		
	QFFFF.VN				
	Other	No data			
MSP1		KD	Other		
	KD	7	2		
	Other	5	3	1.29 (0.26)	0.8 (0.5–1.2)
MSP3		K1	3D7		
	K1	4	2		
	3D7	3	0	0.2 (0.65)	0.9 (0.4–1.7)
MSP6		K1	3D7		
	K1	1	2		
	3D7	3	9	0.2 (0.65)	0.8 (0.2–2.7)
MSPDBL1		ALTAIKY	Other		
	ALTAIKY				
	Other	No data			
MSPDBL2		AHQAIRY	Other		
	AHQAIRY	2	6		
	Other	7	3	0.08 (0.78)	0.9 (0.4–2.0)
Rh1		4HN2QN	Other		
	4HN2QN	1	2		
	Other	1	3	0.33 (0.56)	1.5 (0.4–6.0)
Rh2b		156insQ585del	Other		
	156insQ585del				
	Other	No data			
Rh5		HD	YH		
	HD	13	4		
	YH	0	3	*4 (0.046)*	1.3 (1.0–1.7)

italic value indicates a significant increase in the odds of the Rh5 haplotypes changing compared to staying the same between parasite pairs before and after treatment

*Other* represents genes with more than 2 high frequency haplotypes, the dominant allele was compared to all other alleles in the population, *ND* not determined, *No data* insufficient pairs for the analysis <4

### Linkage disequilibrium analysis

**Fig. 1:**
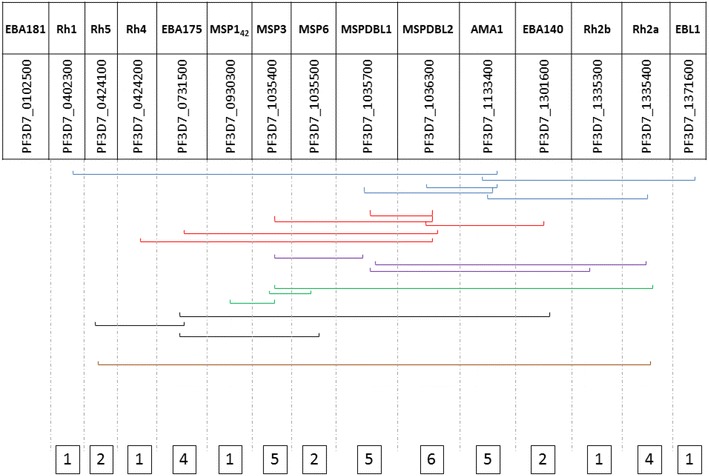
Summary of linkage disequilibrium (LD) analysis depicting 14 merozoite antigens that contain SNPs in LD between genes. The *blues lines* indicate genes with SNPs in LD with *ama1*, *red* those in LD with *mspdbl2*, *purple* *mspdbl1*, *green* *msp3*, *black* *eba175* and *brown* *rh2a* and *rh5* have SNPs in LD with each other. The gene in a *specific column* is in LD with other genes as depicted by the *lines* and the number of genes each gene is in LD with is shown in the *boxes*

A LD analysis was conducted for all genes except *eba181*, which had very few SNPs to analyse. Within gene analysis, all genes contained SNPs in LD except *rh1*, the indels in *msp1, eba175, ebl*-*1* and *rh2b*, the repeat region of *rh4*, SNPs 439 and 442 in *rh5*, SNPs 553 and 716 in *eba140* and multiple SNPs across the *ama1* gene (see Additional file 4). There are potential recombination hot spots between the two LD blocks in *rh2a* at the 5′ and 3′ ends of the sequenced region, the major LD block towards the 5′ end and a smaller LD block towards the 3′ end of *mspdbl2* and the small LD block at the 5′ end followed by a large central LD block and a minor LD block towards the 3′ end of *mspdbl1*. SNPs in LD between genes were identified (see Additional file 4); *mspdbl2*, the gene with the most SNPs (>200) showed the largest number of between gene LD, with SNPs in LD with six other genes, *eba140, eba175, rh4, ama1, msp3* and *mspdbl1*. SNPs in *mspdbl1, msp3* and *ama1* were in LD with five other genes, *rh2a* and *eba175* with four genes, *msp6* and *eba140* with two genes and *ebl*-*1, rh2b, msp1, rh4* and *rh1* with only one gene. Of interest, *rh5* SNP 608 appears to be in LD with *eba175* SNP 1434 and *rh5* SNP 442 with *rh2a* SNPs 8459, 8460, 8463, 8464, and 8465. A summary of the between-gene LD SNP analysis is depicted in Fig. 1.

### Frequency distribution of the merozoite gene polymorphisms in the uncomplicated malaria infections between 2007 and 2008

A large majority of the SNPs were stably maintained over the two-year period. Eight genes contained some SNPs which showed significant (p < 0.05) temporal fluctuations (heterogeneity), Rh1–QN repeat, MSP3–SNPs 219 and 1029, MSPDBL2–SNPs 580 and 587, EBA175- SNPs 1168, 1750 and 1990, AMA1–SNPs 724, 727, 799 and 922 and MSPDBL1 contained a group of about 85 SNPs which were in LD between SNPs 467 and 855, of which SNPs downstream from SNP524 had a p value of 0.008. There were very few SNPs with a p value <0.01, Rh2b–SNP 8149 (192 bp indel), EBA175–SNP1442 and AMA1–SNP1478. Only the Rh5 SNPs 439 and 442 were highly significant, p = 0.00011 (see Additional file 5) and remained significant after a Bonferroni correction.

### Distribution of PfRh5 polymorphisms in all three malaria infection populations

Rh5 codons 48, 197, 203, and 204 were identified in the 11 cultured isolates (see Additional file 6), identifying the previously described codons by Hayton and colleagues [45]. Only codons 197 and 203 were observed in the Kilifi population, however, codon 197 was a singleton SNP in the asymptomatic infections. In the uncomplicated malaria infections, two other rare variants, three DYKNV repeats (codon 132) in two isolates instead of the common 3D7 haplotype of DYKNVDYKNV (two repeats) and a singleton at SNP262 (codon 88) were observed. Furthermore, two additional high frequency SNPs in codons 147 and 148 were identified that were not observed in the laboratory isolates. Codons 147 and 148 were previously described in the MalariaGen data [46, 47]. These codons resulted in two haplotypes, YH and the non-reference 3D7 haplotype HD. A rare combination of YD was also observed in the complicated malaria infections. There were two high frequency, stable haplotypes in the asymptomatic infections YHSY and HDSY with YHSY being dominant over the two-year period from 2008 to 2009. After multiple attempts, we were unable to obtain PCR amplicons from the 2007 samples. The YHY and HDY haplotypes were prevalent and YHY was the dominant and stable haplotype in the complicated malaria infections from 2007 to 2009.

### PfRh5 analysis in all three malaria infection populations

**Fig. 2:**
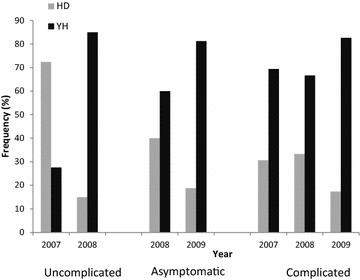
The distribution of Rh5 high frequency haplotypes, YH and HD, in all three malaria infections. The uncomplicated malaria infections from 2007 to 2008 showed a significant shift in haplotype frequency from HD to YH in 2008 (p = 0.00011). However, in the asymptomatic malaria infections between 2008 and 2009, YH was the dominant haplotype in both years and YH was also dominant in the complicated malaria infections from 2007 to 2009

**Fig. 3:**
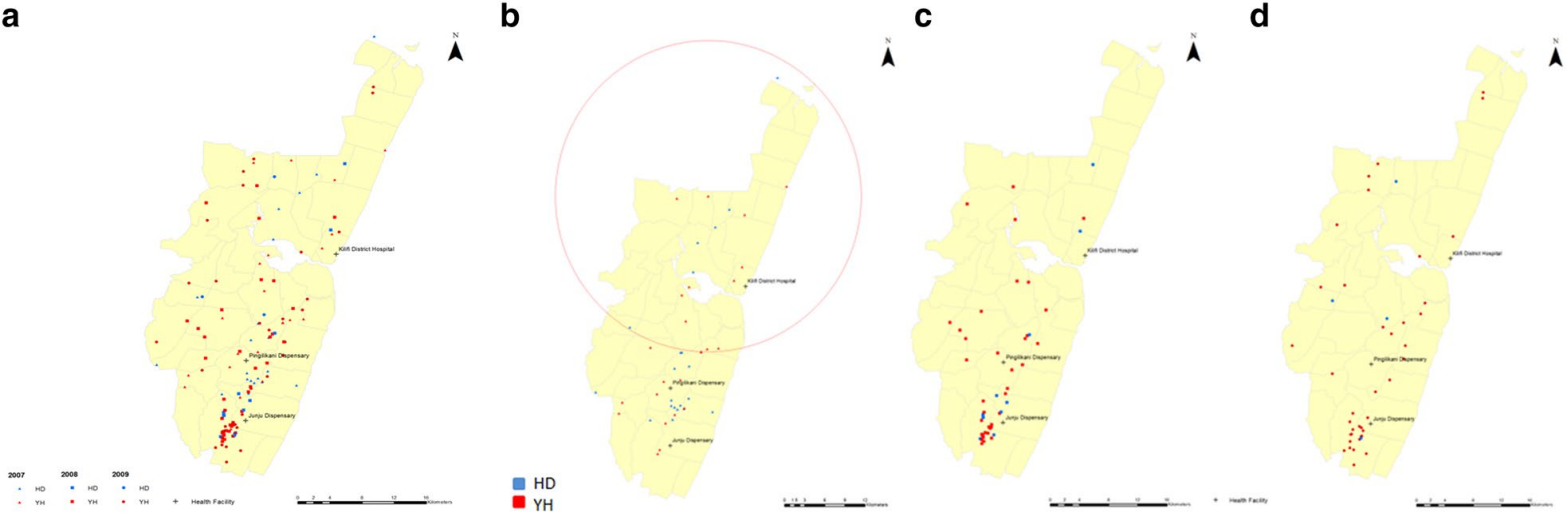
The distribution of PfRh5 YH and HD haplotypes in Kilifi County in the east of Kenya by the Indian Ocean. The *map* highlights the Kilifi health and demographic surveillance system (KHDSS). **a** The distribution of YH and HD haplotypes in all three malaria infection populations (uncomplicated, asymptomatic, complicated) between 2007 and 2009. The years are represented by different *symbols*, *triangle* for 2007, *circle* for 2008 and *square* for 2009. **b** YH and HD haplotype distribution in 2007 showing a significant cluster (depicted by the *red* *circle* showing the region of the cluster) in the north of the KHDSS that contains more YH alleles compared to the parasites from the south (relative risk = 2, p = 0.0024) using the SaTScan software. **c** YH and HD allele distribution in 2008; and, **d** YH and HD haplotype distribution in 2009. The *black* *cross* indicates the health centres Kilifi District Hospital, Junju and Pingilikani dispensaries. The alleles are depicted as *blue* for HD and *red* for YH

The uncomplicated, asymptomatic and complicated malaria infections were compared between the years 2007–2009. Though we excluded mixed infections over this time period, we noticed that the number of mixed infections in the uncomplicated and complicated malaria infections were 6 % (n = 44) and 5 % (n = 150), respectively, about fourfold lower than asymptomatic malaria infections (24 %, n = 54). The analysis focused on the two dominant codons 147 and 148, which showed a highly significant shift in haplotype frequencies between 2007 and 2008 (p = 0.00011), from HD to YH in the uncomplicated malaria infections (Fig. 2). In contrast, there was a dominance of the YH haplotype over the two-year period in the asymptomatic infections (p = 0.19) and over the three-year period (2007–2009) in the complicated malaria infections (p = 0.16). Interestingly, in the uncomplicated malaria infections there was a significant difference between the mean ages 2.5 and 3.5 in 2007 and 2008, respectively (t test unequal, p = 0.0007). However, there was no significant difference in the asymptomatic infections between 2008 and 2009 (mean age 6.4 and 6.6, respectively, t test unequal, p = 0.51) and in the complicated malaria infections (mean age three in all years, t test unequal, p = 0.96). The significant shift in haplotypes in the uncomplicated malaria infections may be due to the significant mean age difference between the years. However, a logistic regression analysis suggested age had no effect on the infecting Rh5 haplotypes, but the chance of being infected with HD parasites decreased with time from 2007 to 2009 and the probability of HD infections increased moving from uncomplicated to asymptomatic malaria infections (Table 2). Also, there were no significant haplotype frequency differences between 2008 and 2009 in uncomplicated and asymptomatic infections. The haplotypes from 2007 to 2009 were plotted on to the KHDSS map (Fig. 3) to examine geographical clustering of haplotypes. Altogether SaTScan did not pick out any significant haplotype (YH or HD) clusters. However, a within-year stratification picked out a significant cluster in the north of the district with more YH infecting parasites when compared to all other possible clusters in 2007 (relative risk = 2, p = 0.0024) (Fig. 3b).

**Table 2:** Logistic regression analysis of Rh5 YH and HD alleles in all three malaria infections between 2007 and 2009

	Haplotype		Odds ratio (95 % CI)	p value
	YH	HD		
Age			1.1 (0.89–1.39)	0.349
Year				
2007	42 (54)	36 (46)	1	
2008	60 (68)	27 (31)	0.11 (0.03–0.37)	<0.001
2009	51 (80)	12 (19)	0.05 (0.01–0.29)	0.001
Population				
Severe	105 (73)	38 (27)	1	
Uncomplicated	25 (51)	24 (49)	4.66 (1.75–12.43)	0.002
Asymptomatic	28 (68)	13 (32)	7.43 (1.57–35.16)	0.011

### Stable Rh5 polymorphisms over a ten-year period of declining malaria transmission

**Fig. 4:**
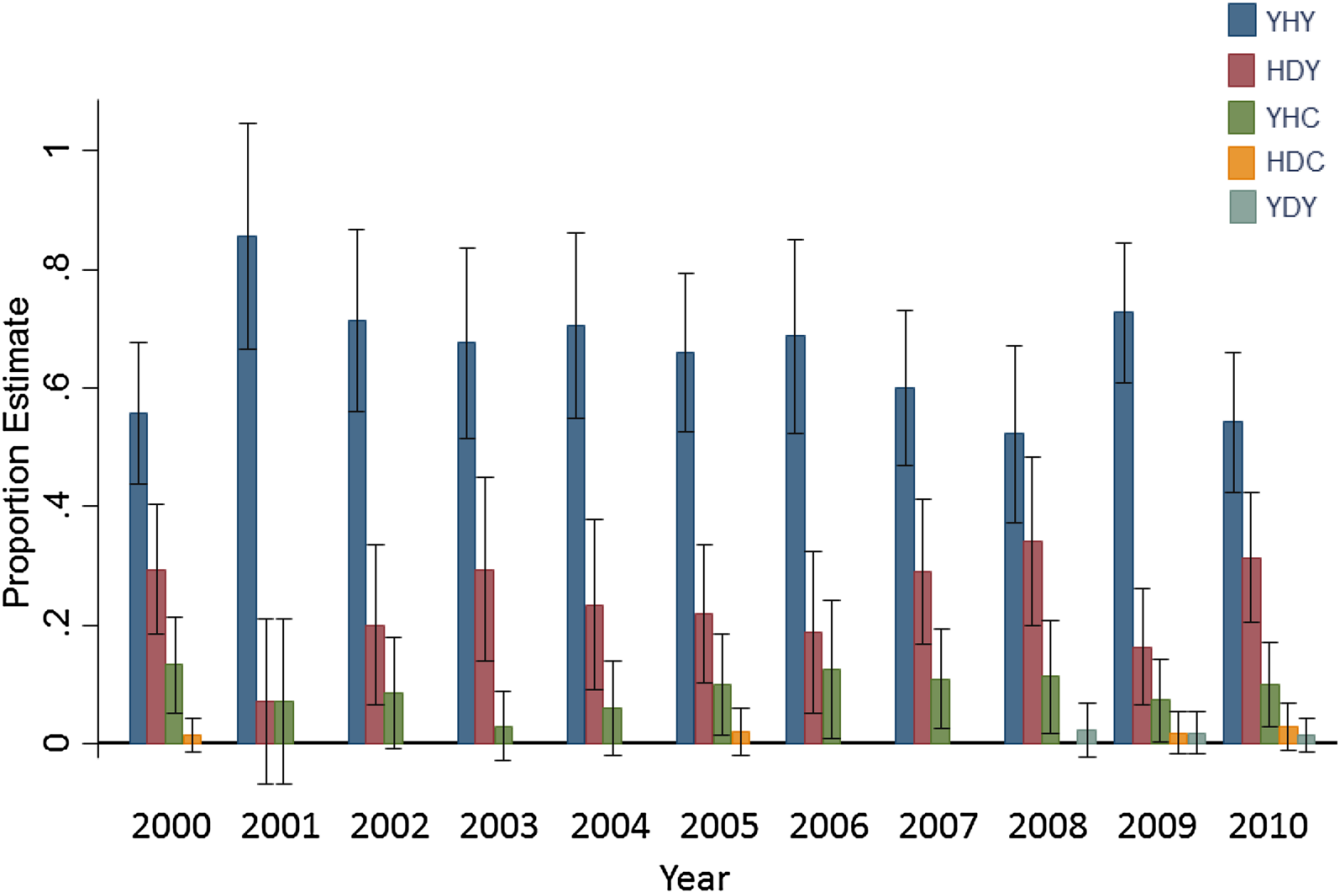
Rh5 haplotype frequency distribution in the complicated malaria infections over a ten-year period. YH was the dominant haplotype from 2000 to 2010

The stable haplotype frequencies of a dominant YHY haplotype, followed by HDY and YHC haplotypes observed between 2007 and 2009, extended over a ten-year period from 2000 to 2010. On occasion rare variants, HDC, were observed in 2000, 2005, 2009 and 2010 and also YDY in 2008, 2009 and 2010 (Fig. 4). There is evidence of a lack of homogeneity (p = 0.0001) and a linear trend (p < 0.001) of an increase in the odds of individuals admitted to KDH coming from the south of the district and from an older age group, >5 years (p = 0.02 and 0.0016, respectively) over the ten-year period. Consequently, the increase in the number of cases from the south is associated with increase in the age of the children admitted to KDH. This population of individuals with complicated malaria had at least one of the severe malaria syndromes of deep breathing, anaemia (haemoglobin <5 g/dl) and cerebral malaria (Blantyre coma score <3) [37]. Over the ten-year period there is evidence of a linear trend of a decrease in the odds of cerebral malaria (p = 0.0004), there is a lack of homogeneity in the odds of population exhibiting deep breathing (p = 0.002), and there were no differences in the individuals with anaemia. However, when location and age were adjusted for in the analysis there were no trends observed in any of the severe malaria syndromes.

## Discussion

The stable and intermediate allele frequencies of all genes except Rh2a and Rh4 (which had only one dominant allele from the region sequenced) are suggestive of these polymorphisms being maintained by stable balancing selection. In particular, the antigens which have previously shown evidence of being under balancing selection in cross-sectional population studies such as AMA1 [11, 48], MSPDBL1 [7], MSPDBL2 [8], and MSP3 [6]. The stable alleles are being maintained by a constant selection pressure on these antigens obtained from parasites, resulting in uncomplicated malaria in children under the age of five over a two-year period.

Though it does not seem to matter if an individual is infected with the YH or HD alleles, what was striking was the shift in allele frequencies observed only in Rh5. This suggests that Rh5 may be under different selection pressures over the two-year period examined, resulting in fluctuating allele frequencies. At an individual level, what appeared to be frequency-dependent selection was observed in the pre- and post-treatment infections in only Rh5. All the changes were from pre-treatment HD infections to YH post-treatment infections. The Rh5 codons 147 and 148 showed a greater likelihood of changing compared to staying the same. In vaccine-induced selection, subsequent infections post-vaccine administration were by parasites of the non-vaccine allele [17, 19]. Thus, similar to the scenario in this study, Rh5 appears to show the same effect of post-treatment infections with predominantly an alternative allele after drug clearance. This variation may be reflected in the Rh5 allele frequencies at a population level, providing the first possible explanation for the observed heterogeneity in Rh5 allele frequencies in the uncomplicated malaria infections. The temporal variation in selection of Rh5 variants may be due to frequency-dependent selection, such that in this population of children with low levels of acquired immunity, the rare YH variants in 2007 have an advantage over the common HD variant and thus YH rises to high frequency in 2008. Furthermore, in the asymptomatic and complicated malaria infections the Rh5 alleles were temporally maintained at stable frequencies, also suggesting frequency-dependent selection. Therefore, at an individual level, allele frequencies may change between infections but at a population level they even out into stable frequencies over time. Since all individuals will eventually encounter all Rh5 variants, the initially rare variant will rise in frequency until equilibrium is attained between all circulating variants.

The second, plausible reason is provided by evidence of differences in clinical outcome, such that children who presented with uncomplicated or asymptomatic malaria were more likely to be infected with HD allele parasites than those with complicated malaria infections. The heterogeneity in selection pressures may therefore be due to differences in the levels of acquired immunity in the individuals with mild and complicated malaria. The variation in the spectrum of disease from asymptomatic to complicated or severe malaria has previously been described and it was primarily attributed to prior exposure and acquired immunity [49].

The third explanation may be as a result of the evidence of spatial patterns of selection in 2007 of a difference in allele frequencies between the north and south of Kilifi County, with more HD infecting parasites in the south compared to the north. These allele frequency differences may be a result of the differences in malaria transmission, which is low in the north and moderate to high in the south [50], and this would alter the levels of immunity of individuals in both regions. A previous study in Senegal, showed higher immune responses to recombinant Rh5 protein in a high malaria transmission area compared to a lower transmission region [51]. Therefore, it is possible that there is selection for more variants in the higher transmission areas and we observed a higher frequency of the HD variant in the south of Kilifi. However, perhaps the missing data from the asymptomatic malaria infections in 2007, also from the south, which was expected from the observed stable frequencies in 2008 and 2009 to be predominantly YH, may have resulted in a sampling bias, resulting in the observed skew in allele frequencies. Nevertheless, the data do provide evidence that fluctuating selection may maintain the variation in Rh5 polymorphisms in the uncomplicated malaria population.

The fourth and more extreme hypothesis is that the fluctuation in Rh5 allele frequencies may be a result of a parasite population bottleneck effect, which greatly reduced the parasite population harbouring the HD allele, perhaps by some variation in transmission intensity that altered the allele frequency over time. However, it may simply be a case of genetic drift in a small localized population that results in the random fluctuations in allele frequencies.

The stable Rh5 allele frequencies in the complicated malaria infections were maintained over a ten-year period on a backdrop of changes in the host population demography. An increase over time of more children coming from the south who are older (>5 years) and a decrease in cerebral malaria cases over time. Suggesting therefore, that even a change over time in the infected host population dynamics does not alter the frequency of Rh5 polymorphisms, perhaps due to the fact that the constant selection pressure is the immune pressure from these individuals. Thus, even though there is a shift to an older age group at a population level, all the individuals still encounter all Rh5 variants, maintaining a stable allele frequency over time.

Overall, the stable and fluctuating Rh5 polymorphism frequencies appear to be the result of the host’s ability to mount an immune response to prevent infection, and it may be the major driving force maintaining the Rh5 polymorphisms in codons 147 and 148. Furthermore, the Rh5 laboratory strain sequences were similar to each other except for the Palo Alto strain, however they differ greatly from the field isolates which contain SNPs in codons 147 and 148. These SNPs were not observed in the laboratory strains.

Recently, the partial Rh5 protein structure has been described [52], which suggests that codons 147 and 148 fall outside the basigin-binding region. They may simply be involved in evading host immune responses and redirecting the immune system away from the important functional binding regions of the protein. Previous studies have demonstrated that Rh5 may be a poorly immunogenic antigen or the responses may be short lived [53, 54] and immune responses are likely to be allele-transcending [47, 54]. Yet, this study potentially suggests that immunity may maintain the high frequency of the polymorphisms in codons 147 and 148. Although the observed allele changes in Rh5 are at a genetic level, the functional impact of the polymorphisms remains to be determined, since cross-strain neutralizing antibodies have previously been demonstrated in vitro [53].

The LD analysis examined the impact of the potential interactions between the 15 merozoite antigens, identifying polymorphic combinations between different genes that may be favoured and maintained by natural selection, potentially due to epistatic selection [55, 56]. Rh5 appears to contain SNPs in LD with SNPs in EBA175 and Rh2a. This interaction between the polymorphisms of different merozoite loci may be due to a functional relationship, providing a fitness advantage such as evading the immune system or invading erythrocytes and emphasizing the overall complex nature of merozoite invasion [57] or immune evasion.

## Conclusions

The shift in allele frequencies at a population level and in individual infections, pre- and post-treatment, seemed to only have an impact on the pattern of Rh5 genetic diversity and codons 147 and 148 may be genetic markers of immune pressure—not to level that results in allele-specific immune responses. However, perhaps an increase in sample size, and an extension of the uncomplicated and asymptomatic malaria parasite population analyses back to 2005 would identify more antigens operating under the same principle of both fluctuating and stable allele frequencies over time. Additionally, the authors note the lack of microsatellite analyses as a limitation, since it would help to confirm unique genotypes and ascertain that the allele frequency changes were limited to the Rh5 locus. Nonetheless, this study provides crucial data describing potential selection forces maintaining Rh5 polymorphisms in the population on a temporal context. It would also be important to determine whether the Rh5 variants elicit an immune response in this genotyped population, to determine the exact mechanism maintaining the polymorphisms in this population.

## Additional files

10.1186/s12936-016-1304-8 PCR conditions and primer sequences for the gene regions analysed in the 15 merozoite genes.
10.1186/s12936-016-1304-8 Merozoite gene allele frequencies in the uncomplicated malaria infections between 2007 and 2008.
10.1186/s12936-016-1304-8 The proportion of same and different genotype parasite pairs *in vivo* and *in vitro*.
10.1186/s12936-016-1304-8 Linkage disequilibrium (LD) analysis of all high frequency (>5%) SNPs in all 15 merozoite genes, except *eba181* due to limited SNP data. The analysis was performed by conducting all possible pair-wise comparisons between all the SNPs across 14 merozoite genes using a pair-wise correlation analysis and a Bonferroni correction for multiple comparisons. The red boxes represent significant correlations between SNPs and thus SNPs in LD, orange represents correlations between SNPs that are not significant, white indicates the SNPs that are not correlated and therefore they are not in LD and grey represents cases where the SNP data is missing in one or both of the paired SNPs.
10.1186/s12936-016-1304-8 SNPs and repeat region frequencies within the 15 merozoite genes from the uncomplicated malaria infections in 2007 and 2008 to identify significant SNPs.
10.1186/s12936-016-1304-8 Rh5 SNPs in 11 geographically distinct laboratory isolates.
