# Supplementary material for: Comparison of allele frequencies of Plasmodium falciparum merozoite antigens in malaria infections sampled in different years in a Kenyan population
Source: Malar J. 2016 May 6;15:261. doi: 10.1186/s12936-016-1304-8 (PMC4858837; doi:10.1186/s12936-016-1304-8)
Supplement: Supplementary file 2 — 10.1186/s12936-016-1304-8 Merozoite gene allele frequencies in the uncomplicated malaria infections between 2007 and 2008. [file 12936_2016_1304_MOESM2_ESM.docx]

Table S1

| **Gene** | **Allele** | **2007 (%)** | **2008 (%)** |
| --- | --- | --- | --- |
| **AMA1** | DDRDFNEY | 5.88 | 5 |
| **(n=17,20)** | DDRDSNEY | 5.88 | 0 |
|  | DDRLLDED | 11.76 | 0 |
|  | DDRLLDEY | 0 | 5 |
|  | DERHFDKY* | 11.76 | 15 |
|  | DQRDFNEY | 0 | 5 |
|  | DQRDLNEY | 5.88 | 0 |
|  | DQRHFDED | 5.88 | 5 |
|  | DQRHFDEY | 11.76 | 0 |
|  | DQRHFDKY | 5.88 | 25 |
|  | NGKDFDED | 5.88 | 5 |
|  | NGRDFNEY | 11.76 | 10 |
|  | NGRDLNED | 11.76 | 5 |
|  | NGRDLNEY | 5.88 | 20 |
| **EBA140** | VSTK | 47.62 | 54.17 |
| **(n=21,24)** | INTK | 38.1 | 20.83 |
|  | VNTK | 9.52 | 4.17 |
|  | INRE | 4.76 | 4.17 |
|  | VNTN | 0 | 4.17 |
|  | VSRE | 0 | 4.17 |
|  | INTN | 0 | 4.17 |
|  | INRK | 0 | 4.17 |
| **EBA181** | NKSFN* | 56 | 50 |
| **(n=9,20)** | NQSFN | 44 | 45 |
|  | NKSFK | 0 | 5 |
| **EBA175** | EEENSKMKK | 0 | 5.88 |
| **(n=5,17)** | EEKKSISENKKI | 40 | 0 |
|  | EEKNPISKNKKK | 0 | 5.88 |
|  | EEKNSISKNKKK | 0 | 5.88 |
|  | EKEKPISENKKK* | 20 | 47.06 |
|  | EKENSKMKK | 0 | 5.88 |
|  | KEENSKMKK | 20 | 0 |
|  | KEKKPISKNKKK | 0 | 5.88 |
|  | KEKKSISENKKK | 0 | 5.88 |
|  | KEKNSISKNKKK | 0 | 5.88 |
|  | KEKNSISKNKNI | 20 | 5.88 |
|  | KEKNSKMKK | 0 | 5.88 |
| **EBL-1** | QFFFF.VN* | 33.33 | 42.11 |
| **(n=6,19)** | QLFLSKS | 33.33 | 26.32 |
|  | .LFLSKP | 33.33 | 15.79 |
|  | .FFFF.VN | 0 | 15.79 |
| **MSP1** | FKFFDD* | 37.14 | 54.55 |
| **(n=35,22)** | FKFFDN | 25.71 | 22.73 |
|  | FFFDD | 11.43 | 13.64 |
|  | FFFDN | 8.57 | 4.55 |
|  | FKSFDD | 8.57 | 4.55 |
|  | FFFHN | 2.86 | 0 |
|  | FKFYDD | 2.86 | 0 |
|  | IFFDN | 2.86 | 0 |
| **MSP3** | K1 | 60 | 57.14 |
| **(n=15,21)** | 3D7* | 40 | 42.86 |
| **MSP6** | 3D7* | 78.95 | 80 |
| **(n=19,25)** | K1 | 21.05 | 20 |
| **MSPDBL1** | AHQAIRY | 54.55 | 10 |
| **(n=11,20)** | ALQAMKY | 36.36 | 25 |
|  | ALTAIKY* | 9.09 | 60 |
|  | AHQAMRY | 0 | 5 |
| **MSPDBL2** | AHQAIRY* | 50 | 42.86 |
| **(n=18,21)** | ALQAIKY | 16.67 | 38.1 |
|  | ALQAMKY | 33.33 | 19.05 |
| **Rh1** | (4xHN)QN* | 30.43 | 19.05 |
| **(n=23,21)** | (4xHN)(2xQN) | 21.74 | 19.05 |
|  | (3xHN)(2xQN) | 13.04 | 23.81 |
|  | (3xHN)QN | 13.04 | 0 |
|  | (5xHN)QN | 4.35 | 9.52 |
|  | (5xHN)(2xQN) | 4.35 | 9.52 |
|  | (4xHN)(3xQN) | 0 | 9.52 |
|  | (5xHN)(3xQN) | 0 | 4.76 |
|  | (5xHN)(4xQN) | 4.35 | 0 |
|  | (6xHN)(2xQN) | 0 | 4.76 |
|  | (6xHN)QN | 4.35 | 0 |
|  | (8xHN)QN | 4.35 | 0 |
| **Rh2a** | KAKQQR* | 93 | 95 |
| **(n=15,20)** | QEERKQK | 7 | 5 |
| **Rh2b** | 156insQ585del | 33.33 | 44.44 |
| **(n=18,9)** | 156ins585del | 16.67 | 11.11 |
|  | 156delQ585ins | 5.56 | 11.11 |
|  | 156delQ585del | 27.78 | 0 |
|  | 192delQ585ins | 16.67 | 0 |
|  | 192delQ585del | 0 | 11.11 |
|  | 192insQ585del | 0 | 22.22 |
| **Rh4** | (IHTNENNINN)(2xEHTNENNINN)(EHTNEKNINN)(EHANEKNIYN)(EHTNENNINY) | 6.7 | 0 |
| **(n=30,19)** | (IHTNENNINN)(EHTNENNINN)(EHTNEKNINN)(EHANEKNINN)(EHTNENNINY) | 3.3 | 0 |
|  | (IHTNENNINN)(EHTNENNINN)(EHTNEKNINN)(EHANEKNIYN)(EHTNENNINY)* | 76.7 | 94.7 |
|  | (IHTNENNINN)(2xEHTNEKNINN)(EHANEKNIYN)(EHTNENNINY) | 6.7 | 0 |
|  | (IHTNENNINN)(EHTNENNINN)(EHTNEKNINN)(EHANEKNIYN)(2xEHTNENNINY) | 3.3 | 0 |
|  | (2xIHTNENNINN)(EHTNENNINN)(EHTNEKNINN)(EHANEKNIYN)(EHTNENNINY) | 3.3 | 0 |
|  | (IHTNENNINN)(EHTNENNINN)(EHTNEKNINN)(EHSNEKNIYN)(EHTNENNINY) | 0 | 5.3 |
| **Rh5** | D(2xDYKNV)HDY | 3.5 | 0 |
| **(n=29,20)** | N(2xDYKNV)HDY | 68.9 | 15 |
|  | N(2xDYKNV)YHC* | 3.5 | 10 |
|  | N(2xDYKNV)YHY | 24.1 | 65 |
|  | N(3xDYKNV)YHY | 0 | 10 |

*3D7 reference allele, 3D7 reference allele for EBA140 is INKK and Rh2b is 156insQ585ins both were not observed in this population, Rh2a haplotype was based on SNPs 8149, 8150, 8151, 8155, 8162, 8168, 8173, 8181 and 8186, n indicates the number of samples per year 2007,2008.
