# Supplementary material for: Comparison of allele frequencies of Plasmodium falciparum merozoite antigens in malaria infections sampled in different years in a Kenyan population
Source: Malar J. 2016 May 6;15:261. doi: 10.1186/s12936-016-1304-8 (PMC4858837; doi:10.1186/s12936-016-1304-8)
Supplement: Supplementary file 3 — 10.1186/s12936-016-1304-8 The proportion of same and different genotype parasite pairs in vivo and in vitro. [file 12936_2016_1304_MOESM3_ESM.docx]

**Table S2**

| **Gene** | **Haplotypes/SNPs** | **Pre- & Post-treatment, n (%)** |
| --- | --- | --- |
| **Rh1** | same | 3 (43) |
|  | different | 4 (57) |
| **Rh2a** | same | 4 (44) |
|  | different | 5 (56) |
| **Rh2b** | same | 0 |
|  | different | 3 (100) |
| **Rh4** | same | 15 (68) |
|  | different | 7 (32) |
| **Rh5** | same | 14 (70) |
|  | different | 6 (30) |
| **EBA140** | same | 7 (47) |
|  | different | 8 (53) |
| **EBA181** | same | 6 (46) |
|  | different | 7 (54) |
| **EBL1** | same | 0 |
|  | different | 1 (100) |
| **EBA175** | same | 0 |
|  | different | 9 (100) |
| **MSP3** | same | 2 (15) |
|  | different | 11 (85) |
| **MSP6** | same | 5 (36) |
|  | different | 9 (64) |
| **MSPDBL1** | same | 0 |
|  | different | 3 (100) |
| **MSPDBL2** | same | 0 |
|  | different | 18 (100) |
| **AMA1** | same | 0 |
|  | different | 20 (100) |
| **MSP1** | same | 0 |
|  | different | 16 (100) |
