# Supplementary material for: Comparison of allele frequencies of Plasmodium falciparum merozoite antigens in malaria infections sampled in different years in a Kenyan population
Source: Malar J. 2016 May 6;15:261. doi: 10.1186/s12936-016-1304-8 (PMC4858837; doi:10.1186/s12936-016-1304-8)
Supplement: Supplementary file 6 — 10.1186/s12936-016-1304-8 Rh5 SNPs in 11 geographically distinct laboratory isolates. [file 12936_2016_1304_MOESM6_ESM.docx]

Table S4

|  | **SNP142** | **SNP590** | **SNP608** | **SNP611** |  |
| --- | --- | --- | --- | --- | --- |
| **Isolate** | **codon 48** | **codon 197** | **codon 203** | **codon 204** | **Haplotype** |
| 3D7 | G | C | G | T | ESCI |
| D10 | G | C | A | T | ESYI |
| FCC2 | G | C | A | T | ESYI |
| HB3 | G | C | A | T | ESYI |
| K1 | G | C | A | T | ESYI |
| PaloAlto | A | C | A | G | KSYK |
| Wellcome | G | A | A | T | EYYI |
| Dd2 | G | C | G | T | ESCI |
| IT | G | A | A | T | EYYI |
| RO33 | G | C | A | T | ESYI |
| V1S | G | A | A | T | EYYI |
